# Alcohol Consumption and Adiposity: A Longitudinal Analysis of 45,399 UK Biobank Participants

**DOI:** 10.3390/ijerph191911945

**Published:** 2022-09-21

**Authors:** Elif Inan-Eroglu, Bo-Huei Huang, Mark Hamer, Annie Britton, Emmanuel Stamatakis

**Affiliations:** 1 Charles Perkins Centre, Faculty of Medicine and Health, School of Health Sciences, The University of Sydney, Sydney, NSW 2050, Australia; 2 Department of Molecular Epidemiology, German Institute of Human Nutrition Potsdam-Rehbruecke, 14558 Nuthetal, Germany; 3 Division of Surgery and Interventional Science, Faculty of Medical Sciences, University College London, London WC1E 6BT, UK; 4 Institute of Epidemiology and Health Care, University College London, London WC1E 6BT, UK

**Keywords:** alcohol, adiposity, body mass index, body fat percentage, waist circumference, waist to hip ratio

## Abstract

The evidence on the association between alcohol consumption and adiposity is inconsistent and fragmented. We investigated the longitudinal association between alcohol consumption pattern and four different adiposity markers with repeated measures of adiposity and obesity incidence. We categorized current drinkers based on the sex-specific quartiles of their weekly alcohol consumption and the UK alcohol drinking guidelines. We used multivariable adjusted generalised linear models. With the exception of a direct association between alcohol volume and body fat percentage (BF%) in women (B = 0.42%; 95%CI: 0.04, 0.80% for women in the top quartile), we found no associations between alcohol consumption and adiposity markers for either sex. Red wine and champagne/white wine consumption were inversely associated with waist circumference (WC) for both sexes (B = −0.58 cm, 95%CI: −0.77, −0.38 cm and B= −0.49 cm, 95%CI: −0.68, −0.29 cm, respectively, for women; B = −0.28 cm, 95%CI: −0.47, −0.08 cm and B = −0.23 cm, 95%CI: −0.42, −0.04 cm, respectively, for men). Female and male spirit drinkers had higher WC than non-spirit drinkers. Alcohol consumption was associated with a lower risk of obesity incidence in women (OR:0.60, 95%CI:0.45, 0.80 for the 2nd quartile, OR:0.53, 95%CI: 0.40, 0.70 for the 3rd quartile and OR:0.61, 95%CI:0.46, 0.80 for the 4th quartile). We found limited evidence of longitudinal associations between alcohol intake and adiposity. The few statistically significant associations we observed are unlikely to be of clinical importance.

## 1. Introduction

Both alcohol consumption and adiposity can increase the risk of many chronic diseases including cancer, type 2 diabetes and cardiovascular diseases [1,2,3,4]. Alcohol has a relatively high energy content (7.1 kcal/g), and calorie intake from alcohol consumption supplements total calorie intake, rather than substituting calories from food, by increasing positive energy balance through stimulating appetite and impairing satiety. Therefore, alcohol consumption may be related to the development of overweight and obesity [5,6]. However, the empirical evidence base on the associations between alcohol consumption and adiposity is inconclusive. Our knowledge about the association between alcohol consumption and adiposity comes from cross-sectional observational studies with contradicting results [7,8,9]. In addition, very few studies have looked at the longitudinal association between alcohol intake and adiposity markers. Yet these studies have also produced conflicting results with several studies finding no association [10,11], a negative association [12], or positive association [13,14,15] between alcohol intake and changes in measures of adiposity.

The interpretation of such results is likely affected by how alcohol intake is measured and classified. While some studies identified drinker types by the daily amount they consume (e.g., alcohol intake above 30g/day as ‘heavy’ drinkers) [16,17], others used frequency in addition to amount per drinking occasion [12,18]. Several studies also measured alcohol consumption from food frequency questionnaires [14]. Making comparisons between studies is difficult due to the variation of adiposity markers across studies. For instance, the majority of studies focused on body mass index (BMI) and body weight [13,16,17], while others used waist circumference (WC) [12,18]. Few studies used multiple adiposity outcomes, mainly a combination of BMI and WC [18]. Confounding effects of socioeconomic status and dietary quality were not accounted for in some of these studies [14,17]. Different types of alcoholic drinks may have different effects on adiposity. For example, beer and spirit consumption promote weight gain, beer consumption promotes abdominal fat distribution whereas wine consumption has no effect or even an inverse effect [18]. In addition, alcohol metabolism may differ by sex that reflects differences in adiposity markers between men and women [19].

In our previous cross-sectional UK Biobank study of the association between alcohol consumption and adiposity indices (BMI and body fat percentage (BF%)), we found that BMI was inversely related to overall alcohol consumption whereas there was no association between BF% and alcohol consumption [20]. The aim of the present study was to investigate the longitudinal association between alcohol consumption patterns and four different adiposity markers including BMI, BF%, WC and waist to hip ratio (WHR) as well as overweight and obesity incidence using a large population-based UK cohort with repeated measures of adiposity.

## 2. Materials and Methods

This research was conducted using the UK Biobank Resource under Application Number 25813. The UK Biobank is a large, population-based cohort study. Around 9.2 million invitations were mailed to recruit 502,616 adults (response rate 5.5%) aged 40–69 years between 2006 and 2010 from 22 centres across the UK to reflect a diverse socioeconomic demographic and mixture of urban and rural residents. Two re-visits took place between 2012 and 2018. Detailed study methods have been published elsewhere [21]. All participants provided informed consent and ethical approval was provided by the National Health Service, National Research Ethics Service (Ref 11/NW/0382). The present study included participants attending both the baseline visit (2007–2010) and at least one re-visit. In the present study, we excluded participants with missing/unusable data at either baseline or baseline alcohol consumption. Participants with at least one repeated measurement of the specific adiposity outcome and baseline alcohol consumption data were included in the study (*n* = 45,399). Figure S1 presents the flow chart of participants included in this research.

### 2.1. Outcomes

All adiposity outcomes were measured at both baseline and follow-up. We calculated BMI from the participant’s weight (kg) and height (m^2^) which were measured by trained staff. BF% was measured by bioimpedance using the Tanita BC−418MA device (Tanita, Tokyo, Japan). Validity of bioelectrical impedance analysis has been shown previously [22]. Waist and hip circumferences were measured by a trained professional using a Wessex non-stretchable sprung tape with the participant in the resting-standing position. WHR was computed as the quotient of WC and hip circumference. This measurement has been used and validated in large health studies (e.g., the BRIGHT hypertension study [23]).

For overweight and obesity incidence, a BMI ≥ 25 kg/m^2^ was considered overweight, and ≥30 kg/m^2^ was considered obese.

### 2.2. Alcohol Consumption

A self-administered touch-screen questionnaire was used to collect the baseline alcohol consumption data. UK Biobank questions are standard version, used in many population studies. The alcohol consumption questionnaire in the UK Biobank has face validity. Participants were asked to classify their current alcohol drinking status as never, previous, or current. Figure S2 describes the categorization of alcohol consumption. Current drinkers were asked additional questions regarding their average weekly/monthly consumption of alcoholic drink types, such as “In an average week/month, how many glasses of red wine would you drink?” We calculated the level of overall alcohol consumption as the number of UK units of alcohol (10 mL/unit) consumed per week; the sum of average weekly intake of red wine; champagne and white wine; beer and cider; spirits; fortified wine; and other alcoholic drinks. We categorized current drinkers based on the sex-specific quartiles of their weekly alcohol consumption and current non-drinkers served as a reference group. Among occasional drinkers (Figure S2), the sex-specific average amount of weekly alcohol consumption was assigned to those with a missing value (2.3 UK units/wk for men and 1.3 UK units/wk for women). For type-specific drinking, participants were dichotomized based on whether they reported any current drinking of a specific type of alcohol (i.e., red wine, champagne, beer and cider, spirits, or fortified wine). This alcohol measurement has been associated with mortality in the UK Biobank [24].

### 2.3. Baseline Covariates

Physical activity (PA) was quantified using the short-form International Physical Activity Questionnaire (IPAQ) [25]. Metabolic Equivalent Task (MET)-minutes of PA/week was calculated by multiplying the MET value of activity by the number of minutes/week. PA was then classified as inactive (<600 MET min/week), active at the lower PA guideline (≥600 MET min/week), or active at the upper PA guideline range (≥1200 MET min/week). We adjusted for chronic illness using a dichotomous variable denoting the presence/absence of major cardiovascular disease (ICD-10 codes I00 to I99) or cancer (C00 to C97, excluding ill-defined, secondary, or unspecified neoplasms). Because using dietary energy intake as a proxy for dietary quality would result in a substantially smaller sample size, we used fruit and vegetable consumption (servings/day) as a proxy for dietary quality. Participants were asked to report the number of servings of cooked vegetables, salad and raw vegetables, fresh fruit, and dried fruit they consumed each day. For example, “On average how many heaped tablespoons of salad or raw vegetables would you eat per day?” One piece of fruit, such as a banana, or one heaped tablespoon of vegetables was considered one serving. Sleep was dichotomized based on participants’ responses to the question “About how many hours sleep do you get in every 24 h? (including naps)”, with participants whose sleep duration was within 7–9 h/day being classified as “adequate”. Sedentary behaviour was calculated by summing the total time spent watching television, using a computer screen or driving. Participants with an implausible sum (>24 h/day) of sedentary time, sleep, and PA were excluded from analysis. Smoking status comprised three categories: never, previous, and current smokers. We used the Townsend deprivation index as an indicator of socioeconomic status, which assigns each participant a score relative to the output area (the smallest UK census area) in which their postcode was located, with higher scores indicating greater socioeconomic deprivation [26].

### 2.4. Statistical Analysis

We calculated Spearmen’s correlation coefficients to determine the consistency of the alcohol consumption between baseline and follow-up. Descriptive statistics were presented with stratification by quartiles of alcohol consumption. We applied a generalised linear model to investigate the association between baseline alcohol consumption status (categorical: quartiles of alcohol consumptions, or binary: type-specific drinking status) and adiposity markers (BMI, BF%, WC, WHR) at follow-up with three levels of adjustment (Models 1–3). We chose the current non-drinkers category as a reference. We adjusted analyses for age, length of follow-up, fruit and vegetable consumption, socioeconomic status, sleep, major illness, physical activity (PA) [27], smoking status and sedentary behaviour. We first adjusted the model for baseline adiposity indicator, age, and follow-up time (Model 1), and additionally adjusted it for socioeconomic status, smoking status, major illness, and sleep (Model 2). In the final model (Model 3), we further adjusted the model for behaviours that directly influence energy balance, i.e., PA, sedentary behaviour, and fruit and vegetable consumption. We also investigated alcoholic drink types (consuming alcohol type vs. not consuming alcohol type (referent)) and adiposity by using Model 3 adjustment. We stratified all analyses by sex. We tested for statistical interaction by entering an alcohol consumption*sex term in the generalised linear fully adjusted model.

We carried out four sets of sensitivity analyses:

(a) Repeated all main models in the sub-sample of participants who had data across all adiposity outcomes (*n* = 17,443).
(b) Repeated all main models after excluding underweight (BMI < 18.5 kg/m^2^) participants at baseline as underweight status might reflect undiagnosed chronic illness.
(c) Repeated all main models by adjusting for energy intake instead of vegetable and fruit consumption as a proxy for dietary quality.
(d) Repeated all main models after excluding non-current drinkers and using 1st quartile of alcohol consumption as a reference.
(e) Repeated all main models with an alternative alcohol categorization we have used before [25] that is based on lifetime drinking status and the UK alcohol consumption guideline (never drinkers, previous drinkers, occasional drinkers, current drinkers within guideline (<14 UK unit/week), within doubled guideline (14 ≤ 28 UK unit/week), and above doubled guideline (≥28 UK unit/week).
(f) Multiple logistic regression models to examine the associations between total alcohol consumption and type-specific alcohol consumption with incidence of overweight (BMI ≥ 25.0 kg/m^2^) and obesity (BMI ≥ 30.0 kg/m^2^). For this analysis, we excluded participants with baseline overweight or obesity. We adjusted the model for baseline age, socioeconomic status, smoking status, major illness, sleep, PA, sitting time, and fruit and vegetable consumption. In type-specific analyses, models were further adjusted for total alcohol consumption.

All the models were two-sided, performed using SAS 9.4 software.

## 3. Results

A total of 40,696 participants were included in the BMI analyses, 18,480 participants in the BF%, 40,790 participants in the WC, and 18,488 participants in the WHR analyses (Figure S1). Table 1 shows alcohol consumption quartile-specific baseline characteristics of the 45,399 participants who were included in any of the above analyses by sex. The mean age was 56.2 ± 7.6 years and 51% of the participants were women. At baseline, 5.0% of the sample reported not currently drinking any alcohol. Compared to men, women had higher BF% (35.6 ± 6.7% and 24.5 ± 5.6%, for women and men, respectively), but lower BMI (26.2 ± 4.7 vs. 27.3 ± 3.9 kg/m^2^, for women and men, respectively), WC (82.2 ± 11.5 vs. 95.1 ± 10.7 cm, for women and men, respectively), and WHR (0.80 ± 0.1 vs. 0.92 ± 0.1, for women and men, respectively). On average, the BMI of the participants changed minimally over time (baseline, 26.7 ± 4.4 kg/m^2^ vs. follow up, 26.8 ± 4.5 kg/m^2^) whereas there was an increase in BF%, WC and WHR (baseline, 30.2 ± 8.3% vs. follow up, 31.1 ± 8.2%; baseline, 88.5 ± 12.9 cm vs. follow up, 89.2 ± 12.9 cm; and baseline, 0.86 ± 0.1 vs. follow up 0.88 ± 0.1, respectively).

There was a strong correlation between baseline and follow up alcohol consumption (Spearman’s *ρ* = 0.84, *p* < 0.001) (Figure S3). Additionally, we calculated stability of alcohol consumption of participants (Table S1) and found that 65% of participants remained stable across all categories.

There was a statistically significant interaction between alcohol consumption*sex for BMI (*p* < 0.0001), BF% (*p* = 0.001), and WC (*p* < 0.0001) as well as alcohol consumption (types of drinks)*sex interaction between WC and red wine (*p* = 0.020).

### 3.1. Overall Alcohol Consumption Volume

#### 3.1.1. General Adiposity

**Figure 1:**
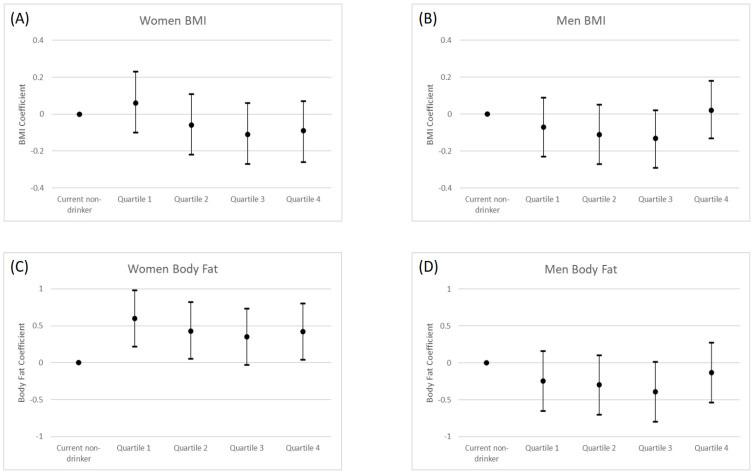
(**A**–**D**) Longitudinal associations between alcohol consumption and general adiposity by sex in the UK Biobank (Model 3). The coefficients were given in the *Y*-axis for each adiposity marker and display the mean difference between the reference category (current non-drinkers) and the other consumption categories. Model 3 is adjusted for baseline age, specific adiposity marker, follow up time (years), Townsend Deprivation Index, smoking status, major illness, sleep duration, PA, sitting time, daily vegetable and fruit consumption. Body mass index (BMI) = Weight (kg)/height (m^2^). BF% was measured by bioimpedance using the Tanita BC-418MA device (Tanita, Tokyo, Japan). Alcohol consumption units by quartile: for women, 1st quartile: ≤1.3 unit; 2nd quartile: <6.9 unit; 3rd quartile: <14.3 unit; 4th quartile: ≥14.3 unit; for men, 1st quartile: ≤6.5 unit; 2nd quartile: <15.9 unit; 3rd quartile: <29.2 unit; 4th quartile: ≥29.2 unit.

Table 2 shows the association between baseline alcohol consumption and BMI and BF% at follow up. Figure 1 presents the association between alcohol consumption and BMI and BF% in the fully adjusted model.

We did not find any association between alcohol consumption and BMI for either sex (Table 2 and Figure 1A,B). Repeating the analysis in the subsample of participants who had data for all adiposity outcomes (*n* = 17,979) did not appreciably change the results (Table S2). Excluding underweight participants, adjusting dietary energy intake instead of vegetable and fruit consumption, and repeating the analysis with the alternative UK guidelines-based alcohol classification produced similar results with the main analysis (Tables S3 and S4 and Figure S4A,B). The sensitivity analysis excluding non-current drinkers and using 1st quartile as a referent showed that female drinkers in the 2nd, 3rd and 4th quartile had lower BMI than light drinkers (1st quartile), although we found little evidence for dose response (Table S5).

We found a direct association between BF% and alcohol consumption in women showing that participants drinking alcohol had higher BF% than current non-drinkers drinkers (GLM coefficient 0.42%; 95% CI: 0.04 to 0.80% for the women in the top quartile) (Table 2 and Figure 1C). In the sensitivity analysis limited to participants with complete data for all adiposity outcomes, we observed similar results (Table S2). In the sensitivity analysis where we excluded underweight participants, we found similar results to the main analysis (Table S3). Adjusting dietary energy intake instead of vegetable and fruit consumption produced similar results with the main analysis (Table S4). We found a similar direct association between alcohol consumption and BF% in the sensitivity analysis with the alternative UK guidelines-based alcohol classification for women (GLM coefficient 0.36%, 95% CI: −0.12 to 0.84% for within-guideline drinkers and GLM coefficient 0.34%, 95% CI: −0.17 to 1.84% for double-the-guidelines drinkers) (Figure S4C). After the exclusion of non-current drinkers and using 1st quartile as a referent, women in the 3rd quartile (GLM coefficient −0.26%; 95% CI: −0.49 to 0.02%) had lower BF% than women in the 1st quartile in our sensitivity analysis (Table S5).

#### 3.1.2. Central Adiposity

**Figure 2:**
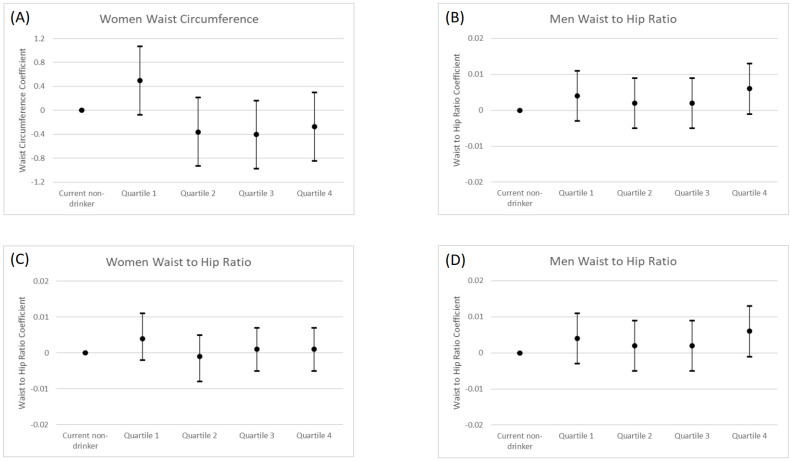
(**A**–**D**) Longitudinal associations between alcohol consumption and central adiposity by sex in the UK Biobank (Model 3). The coefficients were given in the *Y*-axis for each adiposity marker and display the mean difference between the reference category (current non-drinkers) and the other consumption categories. Model 3 is adjusted for baseline age, specific adiposity marker, follow up time (years), Townsend Deprivation Index, smoking status, major illness, sleep duration, PA, sitting time, and daily vegetable and fruit consumption. WC and hip circumference were measured by a trained professional by using flexible plastic tape with the participant in the resting-standing position. Alcohol consumption units by quartile: for women, 1st quartile: ≤1.3 unit; 2nd quartile: <6.9 unit; 3rd quartile: <14.3 unit; 4th quartile: ≥14.3 unit; for men, 1st quartile: ≤6.5 unit; 2nd quartile: <15.9 unit; 3rd quartile: <29.2 unit; 4th quartile: ≥29.2 unit.

Table 2 and Figure 2 present the association between alcohol consumption and WC and WHR at follow up. There were no associations between alcohol consumption and WC and WHR for either sex, either in the main analyses (Figure 2A–D) or the sensitivity analyses (Tables S4, S6 and S7; Supplementary Figure S5A–D). Our sensitivity analysis where we excluded non-current drinkers and used the bottom quartile as a referent showed that women in the 2nd, 3rd and 4th quartile had lower WC than women in the 1st quartile, although there was no evidence for dose-response; whereas men in the top quartile had higher BF% (GLM coefficient 0.41%; 95% CI 0.07 to 0.75%) than men in the 1st quartile (Table S5).

### 3.2. Individual Types of Drinks

#### 3.2.1. General Adiposity

Table 3 presents the associations between individual types of alcoholic drink consumptions and general adiposity. There were weak inverse associations between red wine and BMI at follow up for both sexes (GLM coefficient −0.09 kg/m^2^, 95% CI: −0.15 to −0.04 kg/m^2^ for women and GLM coefficient −0.06 kg/m^2^, 95% CI: −0.11 to −0.02 kg/m^2^ for men). We also found an inverse association between champagne/white wine consumption and BMI in women (GLM coefficient −0.08 kg/m^2^, 95% CI: −0.13 to −0.02 kg/m^2^). Men red wine drinkers had lower BF% compared to non-wine drinking counterparts (GLM coefficient −0.16%, 95% CI: −0.29 to −0.03%).

In the sensitivity analysis excluding underweight participants and adjusting dietary energy intake instead of vegetable and fruit consumption, no appreciable differences was found compared with the main analysis (Tables S8 and S9).

#### 3.2.2. Central Adiposity

Women red wine, champagne/white wine and beer drinkers had lower WC compared to never drinkers of each type of alcoholic drink (GLM coefficient −0.58 cm, 95% CI: −0.77 to −0.38 cm; GLM coefficient −0.49 cm, 95% CI: −0.68 to −0.29 cm and GLM coefficient −0.35 cm, 95% CI: −0.59 to −0.10, respectively) (Table 3). Women spirit drinkers had higher WC compared to never spirit drinkers (GLM coefficient 0.26 cm, 95% CI: 0.04 to 0.49). Men red wine, champagne/white wine and beer drinkers had lower WC compared to never drinkers of each type of alcoholic drink (GLM coefficient −0.28 cm, 95% CI: −0.47 to −0.08, GLM coefficient −0.23 cm, 95% CI: −0.42 to −0.04 and GLM coefficient −0.29 cm, 95% CI: −0.48 to −0.09, respectively), whereas men spirit drinkers had higher WC compared to never spirit drinkers (GLM coefficient 0.21 cm, 95% CI: 0.02 to 0.41 cm) (Table 3). We did not find an association between individual types of alcohol drink consumption and WHR in either sex (Table 3). The results of the sensitivity analysis with the exclusion of underweight participants and the adjustment of dietary energy intake instead of vegetable and fruit consumption were in agreement with the main analysis (Tables S9 and S10).

### 3.3. Incidence of Obesity

Table 4 presents the associations between total alcohol consumption and individual type of alcoholic drink with incident overweight and obesity. A total of 15,512 and 32,867 participants were included in the multiple logistic regression analysis for the association between alcohol consumption and incident overweight and obesity, respectively. About 15% participants developed overweight whereas 5% of participants developed obesity. Although we did not find any association between alcohol consumption and overweight, women drinkers displayed lower odds of incident obesity compared to non-current drinkers (odds ratio: 0.60, 95% CI: 0.45 to 0.80 for the 2nd quartile, odds ratio: 0.53, 95% CI: 0.40 to 0.70 for the 3rd quartile and odds ratio: 0.61, 95% CI: 0.46 to 0.80 for the 4th quartile). There was a curvilinear (U-shape) association of alcohol consumption with incidence of obesity in women. Alcohol consumption was not associated with incidence of overweight or obesity in men. The results of the sensitivity analysis with the adjustment of dietary energy intake were in agreement with the main analysis indicating lower odds of incident obesity in female drinkers compared to non-current drinkers (Table S11). We found that women in the 2nd, 3rd and 4th quartile displayed lower odds of incident obesity compared to light drinkers (1st quartile) (Table S12).

In women, red wine and champagne/white wine consumption were associated with lower risk of overweight (odds ratio: 0.83, 95% CI: 0.73 to 0.94 and odds ratio: 0.82, 95% CI: 0.73 to 0.93, respectively). There was no association between alcohol drink type and incident overweight in men. Red wine and champagne/white wine drinkers had lower risk of overweight and obesity compared to non-drinkers of each type of alcoholic drink for both sexes (Table 4). Women spirit drinkers had higher odds of obesity incidence than non-spirit drinkers (odds ratio: 1.23, 95% CI: 1.05 to 1.44). In addition, men beer drinkers had lower odds of obesity incidence (odds ratio: 0.84, 95% CI: 0.71 to 0.99). The results of the sensitivity analysis with adjustment of dietary energy intake instead of vegetable and fruit consumption were in agreement with the main analysis (Table S11).

## 4. Discussion

We examined the sex-specific longitudinal associations between alcohol volume and type and four common adiposity markers. Our results have practical value as randomised controlled trials on alcohol consumption and long-term health outcomes (including adiposity markers) are neither feasible nor ethical.

A positive association between alcohol consumption and weight gain is biologically plausible for many reasons, including the energy density of alcoholic drinks, stimulation of appetite and a lesser satiation effect of alcohol [5,6]. However, the results of many prospective studies examining the association between alcohol consumption and weight gain do not always support the contribution of alcohol consumption to increased adiposity. Our results are in broad agreement with such studies as we did not find associations between alcohol consumption and BMI for either sex. A previous study among women aged 35 to 47 years also showed that alcohol intake was not associated with subsequent weight gain [27]. Wannamethee et al. [17] also showed in their prospective study of 49,324 women 27 to 44 years old that light to moderate drinking (up to 30 g/d) was not associated with weight gain, whereas heavier drinking may exacerbate weight gain. 

In the cross-sectional analysis of the association between alcohol intake and adiposity of men, Wannamethee et al. [8] showed a significant increase of BF% with increasing alcohol intake. We found a direct association between alcohol consumption and BF% in women, a finding that might be explained by chance due to multiple testing.

Several studies have reported inconsistent results with regard to alcohol consumption and WC [12,18]. We did not find statistically significant associations between alcohol consumption and WC and WHR for either sex. Tolstrup et al. [12] showed in their prospective analysis of a Danish cohort that drinking frequency was inversely associated with changes in WC in women and men, meaning that non-drinkers and the lightest drinkers had the highest odds for major gain in WC. Another large prospective study from the US reported no statistically significant association between WC and alcohol consumption [28].

The impact of alcohol drinking on adiposity markers is affected by complex interrelationships between alcohol consumption and various lifestyle, clinical and physiological factors. For example, female alcohol drinkers tend to substitute alcohol for other foods without increasing total energy intake whereas men add energy from alcohol to their total energy intake [29,30]. Different results between men and women are difficult to explain and may be caused by the biological tendency of men and women to store fat in different parts of the body and specific effects of individual types of alcoholic drinks. Our formal interaction tests showed statistically significant alcohol consumption*sex interactions for BF% and WC, a finding supported by the stratified analyses where we found direct association between alcohol consumption and BF% and WC only in women.

We found weak inverse associations between red wine consumption and BMI for both sexes and weak inverse association between red wine consumption and BF% in men. Sayon-Orea et al. [31] showed in a Mediterranean cohort that wine consumption was not associated with yearly weight change. The inverse association between wine consumption and BMI could also be attributed to the healthier dietary and lifestyle habits of wine drinkers [32]. We did not find an association between spirits or beer consumption and BMI for either sex. In contrast, Sayon-Orea et al. [31] found that spirits and beer consumption were associated with weight gain. Wannamethee et al. [17] also found that the lower weight gain in light and moderate drinkers was seen in both beer and wine drinkers but not in liquor drinkers.

We found an inverse association of red wine and beer consumption with WC for both sexes and between champagne/white wine consumption and WC in women, whereas we did not find an association between alcohol consumption and WHR for either sex. We also showed that spirit drinkers had higher WC than never spirit drinkers, but the magnitude of coefficients were small. In the Copenhagen City Heart Study, Vadstrup et al. [33] showed that moderate to high consumption of spirits was associated with high WC in both men and women, a finding questioned by the authors themselves due to lack of precision of estimates. The same study found an inverse association between wine consumption and WC. In their prospective study, Halkjær et al. [34] found a U-shaped association between alcohol from wine and differences in WC for both sexes and a positive association of alcohol from spirits with differences in WC in women. In contrast to our study, Schutze et al. [35] showed a positive association in men and no association in women between beer consumption and WC in their prospective study. Sex-specific differences in the metabolism of alcohol may explain these results. For instance, female drinkers have a lower activity of alcohol dehydrogenase influencing the degradation of ethanol through a microsomal ethanol-oxidizing system [36].

The weak inverse associations between red wine and champagne/white wine consumption and BMI and WC are biologically implausible and unlikely to be practically meaningful. The absence of consistent detrimental associations in our study might indicate that light to moderate alcohol consumption may be part of a healthy diet. In the context of general health, this idea is refuted by a large 2016 Global Burden of Disease [37] analysis which concluded that alcohol consumption is a leading risk factor for global disease burden and causes substantial health loss, and that there is ‘no safe level of alcohol’.

We found that alcohol consumption was associated with a lower risk of obesity incidence in women. In their prospective cohort of 19,220 US women aged ≥ 39 years, Wang et al. [38] found that compared with non-drinkers, initially normal-weight women that consumed light to moderate amounts of alcohol experienced smaller weight gain and lower risk of becoming overweight or obese. We also found that red wine and champagne/white wine drinkers had lower risk of overweight and obesity compared to non-drinkers of each type of alcoholic drink for both sexes, while there was no association between spirit consumption and the risk of developing overweight and obesity. There were inverse associations between beer consumption and the risk of obesity in men. In contrast, Sayon-Orea et al. [31] showed that beer and spirit consumption was associated with a higher risk of developing overweight/obesity compared with non-drinkers. There were no apparent associations between wine consumption and developing overweight/obesity.

Our study has several notable strengths, including its longitudinal design and repeated measurements. We used a large general population. We had detailed information on both drinking volume and drinking patterns (frequency, amount of alcohol intake and type-specific alcohol consumption). The latter allowed us to undertake type-specific alcohol consumption analyses. We also had detailed information on adiposity markers including BMI, BF%, WC and WHR. Lastly, we were able to control for a large number of possible confounding factors, such as PA, illness, and socioeconomic status. Our study also had some limitations. Firstly, we relied on alcohol self-reports which may lead to reporting biases. However, self-reported alcohol consumption measures, such as the ones used in UK Biobank, have demonstrated adequate reliability and validity [39,40]. The low response rate of the UK Biobank Study (5.5%) may lead to selection bias, although a recent study showed that it is unlikely to influence the direction of the association between alcohol and health outcomes [41].

## 5. Conclusions

In summary, our large-scale longitudinal cohort analysis suggests that alcohol consumption is not associated with increases in common adiposity markers such as BMI, WC and WHR. Alcohol consumption was associated with higher BF% only in women. Red wine and champagne/white wine consumption was inversely associated with WC. Our study does not support the popular belief that calories from alcoholic drinks increase central or general adiposity.

## Figures and Tables

**Table 1:** Baseline characteristics of study sample by sex (*n* = 45,399) *.

	Women					Men				
Drinking Status (UK Units)	Non-Current Drinkers	1st Quartile (≤1.3)	2nd Quartile (<6.9)	3rd Quartile (<14.3)	4th Quartile (≥14.3)	Non-Current Drinkers	1st Quartile (≤6.5)	2nd Quartile (<15.9)	3rd Quartile (<29.2)	4th Quartile (≥29.2)
***n***	1360	5009	4841	5994	6088	899	4712	5571	5708	5217
**Age (years)**	56.0 (7.8)	55.3 (7.6)	55.9 (7.5)	55.6 (7.3)	55.2 (7.2)	56.2 (8.1)	56.4 (8.0)	57.0 (7.7)	57.1 (7.6)	56.9 (7.3)
**Body mass index (kg/m^2^) ^1^**	26.9 (5.5)	27.2 (5.4)	26.1 (4.6)	25.7 (4.3)	26.0 (4.3)	27.2 (4.7)	27.3 (4.3)	26.9 (3.8)	27.1 (3.6)	27.8 (3.8)
**BF% ^2^**	36.2 (7.2)	36.6 (7.0)	35.4 (6.7)	34.9 (6.5)	35.3 (6.5)	24.5 (6.2)	24.4 (5.9)	24.0 (5.5)	24.4 (5.4)	25.3 (5.3)
**WC (cm) ^3^**	83.3 (13.1)	84.0 (12.7)	81.7 (11.4)	80.9 (10.7)	82.0 (10.8)	94.7 (12.2)	95.2 (11.5)	94.1 (10.4)	94.7 (10.0)	96.5 (10.5)
**WHR ^3^**	0.8 (0.1)	0.8 (0.1)	0.8 (0.1)	0.8 (0.1)	0.8 (0.1)	0.9 (0.1)	0.9 (0.1)	0.9 (0.1)	0.9 (0.1)	0.9 (0.1)
**Townsend deprivation index ^4^**	−1.5 (3.0)	−1.7 (2.9)	−2.1 (2.6)	−2.2 (2.5)	−2.0 (2.6)	−1.2 (3.1)	−1.8 (2.8)	−2.3 (2.6)	−2.3 (2.5)	−2.0 (2.7)
**Fruit and vegetable consumption (servings/day)**	4.9 (3.5)	4.7 (3.0)	4.7 (2.8)	4.7 (2.7)	4.7 (3.0)	4.2 (3.9)	3.9 (3.2)	4.0 (2.7)	4.0 (2.9)	3.8 (2.8)
**Sedentary behaviour (hours/day)**	4.0 (2.9)	4.0 (2.9)	4.0 (2.5)	3.9 (2.5)	4.0 (2.7)	4.7 (3.5)	4.9 (3.1)	4.7 (2.9)	4.8 (2.9)	4.9 (3.1)
**CVD/cancer history (%) ^5^**	27.2	25.7	24.1	23.6	23.3	35.0	34.4	32.2	31.6	31.8
**Adequate sleep duration** **(7–9 h) (%)**	71.9	76.7	78.5	78.6	77.6	72.9	76.0	77.5	77.8	76.7
**Never smoker (%)**	75.6	71.3	71.8	65.6	50.1	64.3	66.2	63.7	52.2	38.9
**Meeting physical activity guidelines (%) ^6^**	72.7	72.4	76.6	76.7	76.9	70.4	73.3	77.4	79.5	78.3

Data are presented as mean (standard deviation) or percentage as applicable. Alcohol consumption units by quartile: for women, 1st quartile: ≤1.3 unit; 2nd quartile: <6.9 unit; 3rd quartile: <14.3 unit; 4th quartile: ≥14.3 unit; for men, 1st quartile: ≤6.5 unit; 2nd quartile: <15.9 unit; 3rd quartile: <29.2 unit; 4th quartile: ≥29.2 unit. ^1^ Body mass index = Weight (kg)/height (m^2^). ^2^ BF% was measured by bioimpedance using the Tanita BC-418MA device (Tanita, Tokyo, Japan). ^3^ Waist and hip circumference were measured by a trained professional by using flexible plastic tape with the participant in the resting-standing position. ^4^ Townsend deprivation index scores ranged from −6 to 11. Scores were derived from national census data. Each participant was assigned a score relative to the output area in which their postcode was located. Higher scores reflect a higher degree of socioeconomic deprivation. ^5^ Disease history was based on the ICD10. ^6^ Physical activity (PA) patterns were classified based on the World Health Organization PA guidelines (600 metabolic equivalent minutes per week). * Because sample size varied by analytic outcome, this table presents descriptive features of all participants entered in at least one analysis.

**Table 2:** Longitudinal associations between baseline alcohol consumption and adiposity at follow-up in the UK Biobank.

	General Adiposity								Central Adiposity							
	BMI (*n* = 40,696)				BF% (*n* = 18,480)				WC (*n* = 40,790)				WHR (*n* = 18,488)			
	Model 1		Model 2		Model 1		Model 2		Model 1		Model 2		Model 1		Model 2	
	Coefficient	95% CI	Coefficient	95% CI	Coefficient	95% CI	Coefficient	95% CI	Coefficient	95% CI	Coefficient	95% CI	Coefficient	95% CI	Coefficient	95% CI
**Women**																
Quartile 1	0.06	−0.11, 0.22	0.06	−0.1, 0.23	**0.62**	**0.24, 1**	**0.6**	**0.22, 0.98**	0.48	−0.09, 1.06	0.51	−0.07, 1.08	0.004	−0.002, 0.011	0.004	−0.002, 0.01
Quartile 2	−0.07	−0.24, 0.09	−0.06	−0.22, 0.11	**0.45**	**0.07, 0.84**	**0.43**	**0.05, 0.82**	−0.43	−1, 0.15	−0.36	−0.94, 0.21	−0.001	−0.008, 0.005	−0.001	−0.008, 0.005
Quartile 3	−0.13	−0.29, 0.04	−0.11	−0.27, 0.05	0.38	0, 0.75	0.35	−0.03, 0.73	−0.47	−1.03, 0.09	−0.41	−0.97, 0.16	0.001	−0.005, 0.007	0.001	−0.005, 0.007
Quartile 4	−0.09	−0.25, 0.07	−0.1	−0.26, 0.07	**0.46**	**0.09, 0.84**	**0.42**	**0.04, 0.8**	−0.28	−0.84, 0.29	−0.28	−0.84, 0.29	0.002	−0.005, 0.008	0.001	−0.006, 0.007
**Men**																
Quartile 1	−0.09	−0.25, 0.07	−0.07	−0.23, 0.09	−0.24	−0.65, 0.16	−0.24	−0.65, 0.16	−0.13	−0.76, 0.5	−0.04	−0.67, 0.59	0.003	−0.004, 0.01	0.004	−0.004, 0.011
Quartile 2	−0.15	−0.3, 0.01	−0.11	−0.26, 0.05	−0.31	−0.71, 0.09	−0.3	−0.7, 0.11	−0.48	−1.1, 0.14	−0.34	−0.97, 0.28	0.001	−0.006, 0.008	0.002	−0.005, 0.009
Quartile 3	−0.16	−0.31, 0	−0.12	−0.28, 0.03	−0.38	−0.79, 0.02	−0.39	−0.79, 0.02	−0.38	−1, 0.24	−0.28	−0.91, 0.34	0.001	−0.006, 0.008	0.001	−0.006, 0.008
Quartile 4	0.04	−0.12, 0.19	0.03	−0.12, 0.19	−0.08	−0.48, 0.33	−0.12	−0.53, 0.28	0.37	−0.26, 1	0.36	−0.27, 0.99	0.006	−0.002, 0.013	0.005	−0.002, 0.012

Generalised linear model coefficient; mean differences (in risk factor values) between the reference category (current non-drinker) and each of the other alcohol consumption categories. Alcohol consumption units by quartile: for women, 1st quartile: ≤1.3 unit; 2nd quartile: <6.9 unit; 3rd quartile: <14.3 unit; 4th quartile: ≥14.3 unit; for men, 1st quartile: ≤6.5 unit; 2nd quartile: <15.9 unit; 3rd quartile: <29.2 unit; 4th quartile: ≥29.2 unit. Model 1 is adjusted for baseline age, specific adiposity marker, and follow up time (years). Model 2 is further adjusted for Townsend Deprivation Index, smoking status, major illness, sleep duration (h/night). Body mass index (BMI) = Weight (kg)/height (m^2^). BF% was measured by bioimpedance using the Tanita BC-418MA device (Tanita, Tokyo, Japan). WC and hip circumference were measured by a trained professional by using flexible plastic tape with the participant in the resting-standing position. Bold font indicates statistical significance (*p* < 0.05).

**Table 3:** Longitudinal associations between type-specific alcohol consumption and adiposity at follow up in the UK Biobank (*n* = 39,698).

	Model 1		Model 2		Model 3	
	Coefficient	95% CI	Coefficient	95% CI	Coefficient	95% CI
*BMI (n = 40,696)*						
**Women**						
Red wine	**−0.11**	**−0.16, −0.05**	**−0.09**	**−0.15, −0.04**	**−0.09**	**−0.15, −0.04**
Champagne	**−0.09**	**−0.15, −0.04**	**−0.08**	**−0.13, −0.02**	**−0.08**	**−0.13, −0.02**
Beer	−0.04	−0.11, 0.03	−0.04	−0.11, 0.03	−0.04	−0.11, 0.03
Spirits	0.06	0, 0.13	0.06	−0.01, 0.12	0.06	−0.01, 0.12
Fortified wine	0.01	−0.09, 0.1	0.02	−0.08, 0.12	0.02	−0.08, 0.12
**Men**						
Red wine	**−0.09**	**−0.14, −0.04**	**−0.06**	**−0.11, −0.01**	**−0.06**	**−0.11, −0.02**
Champagne	**−0.07**	**−0.12, −0.03**	−0.05	−0.09, 0	−0.05	−0.09, 0
Beer	−0.02	−0.07, 0.03	−0.02	−0.07, 0.03	−0.02	−0.08, 0.03
Spirits	0.04	−0.01, 0.09	0.03	−0.02, 0.08	0.03	−0.02, 0.08
Fortified wine	−0.02	−0.11, 0.06	−0.01	−0.09, 0.08	−0.01	−0.09, 0.08
*BF% (n = 18,480)*						
**Women**						
Red wine	−0.06	−0.19, 0.07	−0.06	−0.2, 0.07	−0.06	−0.19, 0.08
Champagne	−0.03	−0.17, 0.1	−0.04	−0.17, 0.1	−0.03	−0.17, 0.1
Beer	−0.09	−0.26, 0.08	−0.1	−0.27, 0.08	−0.09	−0.26, 0.09
Spirits	0.16	0, 0.31	0.14	−0.01, 0.3	0.14	−0.01, 0.3
Fortified wine	−0.01	−0.23, 0.21	−0.02	−0.24, 0.2	−0.02	−0.24, 0.2
**Men**						
Red wine	**−0.18**	**−0.31, −0.06**	**−0.17**	**−0.3, −0.05**	**−0.16**	**−0.29, −0.03**
Champagne	**−0.13**	**−0.25, −0.01**	−0.11	−0.23, 0.01	−0.1	−0.22, 0.02
Beer	0.03	−0.1, 0.16	0.02	−0.11, 0.15	0.02	−0.11, 0.15
Spirits	−0.04	−0.16, 0.09	−0.05	−0.18, 0.07	−0.05	−0.18, 0.07
Fortified wine	−0.05	−0.27, 0.17	−0.03	−0.25, 0.19	−0.02	−0.24, 0.2
*WC (n = 40,790)*						
**Women**						
Red wine	**−0.65**	**−0.84, −0.45**	**−0.6**	**−0.8, −0.41**	**−0.58**	**−0.77, −0.38**
Champagne	**−0.55**	**−0.74, −0.36**	**−0.5**	**−0.69, −0.3**	**−0.49**	**−0.68, −0.29**
Beer	**−0.35**	**−0.59, −0.11**	**−0.37**	**−0.61, −0.12**	**−0.35**	**−0.59, −0.1**
Spirits	**0.3**	**0.07, 0.52**	**0.28**	**0.06, 0.5**	**0.26**	**0.04, 0.49**
Fortified wine	−0.26	−0.6, 0.07	−0.21	−0.54, 0.13	−0.19	−0.53, 0.15
**Men**						
Red wine	**−0.4**	**−0.6, -0.21**	**−0.32**	**−0.51, −0.12**	**−0.28**	**−0.47, −0.08**
Champagne	**−0.34**	**−0.53, −0.15**	**−0.25**	**−0.44, −0.06**	**−0.23**	**−0.42, −0.04**
Beer	**−0.28**	**−0.48, −0.08**	**−0.29**	**−0.48, −0.09**	**−0.29**	**−0.48, −0.09**
Spirits	**0.24**	**0.04, 0.43**	**0.22**	**0.02, 0.41**	**0.21**	**0.02, 0.41**
Fortified wine	0.01	−0.33, 0.35	0.08	−0.26, 0.42	0.1	−0.24, 0.44
*WHR (n = 18,488)*						
**Women**						
Red wine	−0.003	−0.005, 0	−0.003	−0.005, 0	−0.002	−0.005, 0
Champagne	−0.002	−0.004, 0.001	−0.003	−0.005, 0	−0.003	−0.005, 0
Beer	0	−0.003, 0.003	−0.001	−0.003, 0.002	0	−0.003, 0.003
Spirits	0.002	−0.001, 0.005	0.002	−0.001, 0.004	0.002	−0.001, 0.004
Fortified wine	−0.001	−0.005, 0.003	0	−0.004, 0.003	0	−0.004, 0.003
**Men**						
Red wine	−0.002	−0.004, 0.001	−0.001	−0.003, 0.001	−0.001	−0.003, 0.001
Champagne	−0.003	−0.005, 0	−0.001	−0.003, 0.001	−0.001	−0.003, 0.001
Beer	−0.001	−0.003, 0.002	−0.001	−0.003, 0.001	0	−0.003, 0.002
Spirits	0.003	0, 0.005	0.003	0, 0.005	0.003	0, 0.005
Fortified wine	0.001	−0.003, 0.004	0.001	−0.003, 0.005	0.001	−0.002, 0.005

Generalised linear model coefficient; mean differences (in risk factor values) between participants who did not consume the relevant alcohol type (the referent) and participants who reported consuming the relevant alcohol type. Model 1 is adjusted for baseline age, specific adiposity marker, and follow up time (years). Model 2 is adjusted for baseline age, specific adiposity marker, follow up time (years), Townsend Deprivation Index, smoking status, major illness, and sleep duration (h/night). Model 3 is adjusted for baseline age, specific adiposity marker, follow up, Townsend Deprivation Index, smoking status, major illness, sleep duration (h/night), PA, sitting time, and daily vegetable and fruit consumption. Body mass index (BMI) = Weight (kg)/height (m^2^). BF% was measured by bioimpedance using the Tanita BC-418MA device (Tanita, Tokyo, Japan). WC and hip circumference were measured by a trained professional by using flexible plastic tape with the participant in the resting-standing position. Bold font indicates statistical significance (*p* < 0.05).

**Table 4:** The associations of total alcohol consumption and individual type of alcoholic drink with incident overweight (BMI ≥ 25 kg/m^2^) and obesity (≥30 kg/m^2^).

	Incident Overweight (BMI ≥ 25 kg/m^2^)				Incident Obesity (≥30 kg/m^2^)			
	Women (Case/Total = 1389/9725)		Men (936/5787)		Women (904/17,077)		Men (864/15,790)	
	Odds Ratio	95% CI	Odds Ratio	95% CI	Odds Ratio	95% CI	Odds Ratio	95% CI
***Total alcohol consumption* ^a^**								
Quartile 1	1.21	0.93, 1.59	0.91	0.64, 1.29	0.93	0.71, 1.22	0.93	0.65, 1.34
Quartile 2	0.89	0.68, 1.17	0.94	0.66, 1.33	**0.6**	**0.45, 0.8**	0.8	0.56, 1.15
Quartile 3	0.83	0.64, 1.09	0.89	0.62, 1.26	**0.53**	**0.4, 0.7**	0.82	0.58, 1.18
Quartile 4	0.94	0.72, 1.23	1.13	0.79, 1.62	**0.61**	**0.46, 0.8**	1.09	0.77, 1.56
***Alcohol type* ^b^**								
Red wine	**0.83**	**0.73, 0.94**	0.9	0.77, 1.06	**0.75**	**0.65, 0.88**	**0.8**	**0.69, 0.94**
Champagne	**0.82**	**0.73, 0.93**	0.88	0.76, 1.03	**0.81**	**0.7, 0.94**	**0.82**	**0.71, 0.95**
Beer	0.94	0.82, 1.09	0.94	0.8, 1.11	0.9	0.76, 1.08	**0.84**	**0.71, 0.99**
Spirits	1.08	0.94, 1.24	1.05	0.9, 1.23	**1.23**	**1.05, 1.44**	1.06	0.92, 1.23
Fortified wine	**0.78**	**0.63, 0.98**	0.83	0.63, 1.1	1.02	0.79, 1.31	1.04	0.8, 1.34

Multiple logistic regression model. Alcohol consumption units by quartile: for women, 1st quartile: ≤1.3 unit; 2nd quartile: <6.9 unit; 3rd quartile: <14.3 unit; 4th quartile: ≥14.3 unit; for men, 1st quartile: ≤6.5 unit; 2nd quartile: <15.9 unit; 3rd quartile: <29.2 unit; 4th quartile: ≥29.2 unit. Model is adjusted for baseline age, Townsend Deprivation Index, smoking status, major illness, sleep duration, PA, sitting time, follow-up time, and daily vegetable and fruit consumption. In type-specific analyses, models were further adjusted for total alcohol consumption. ^a^ Non-current drinkers is the referent group. ^b^ Participants who did not consume the relevant alcohol type is the referent group. Bold font indicates statistical significance (*p* < 0.05).

## Data Availability

The UK Biobank is an open-access resource. Bona fide researchers can apply to use the UK Biobank dataset by registering and applying at http://www.ukbiobank.ac.uk/register-apply/.

## Supplementary Materials

The following supporting information can be downloaded at https://www.mdpi.com/article/10.3390/ijerph191911945/s1, Figure S1: The flow chart of participants, Figure S2: The categorization of alcohol consumption, Figure S3: Correlation between baseline and follow up alcohol consumption, Figure S4: Longitudinal associations between alternative alcohol consumption categorization and general adiposity by sex in the UK Biobank (Model 3), Figure S5: Longitudinal associations between alternative alcohol consumption categorization and central adiposity by sex in the UK Biobank (Model 3); Table S1: Alcohol consumption status change of the UK Biobank participants, Table S2: Longitudinal associations between baseline alcohol consumption and general adiposity at follow up in the UK Biobank of participants without missing adiposity outcomes (*n* = 17,979), Table S3: Longitudinal associations between alcohol consumption and general adiposity at follow-up in the UK Biobank after excluding underweight (<18.5 kg/m^2^) participants at baseline, Table S4: Longitudinal associations between alcohol consumption and general adiposity at follow-up in the UK Biobank adjusting with daily energy intake as a dietary proxy, Table S5: Longitudinal associations between alcohol consumption at baseline and general adiposity at follow-up in the UK Biobank after excluding non-current drinkers, Table S6: Longitudinal associations between baseline alcohol consumption and central adiposity at follow-up in the UK Biobank of participants without missing adiposity outcomes (*n* = 17,979), Table S7: Longitudinal associations between alcohol consumption and central adiposity at follow-up in the UK Biobank after excluding underweight (<18.5 kg/m^2^) participants at baseline, Table S8: Longitudinal associations between type-specific alcohol consumption and general adiposity at follow-up in the UK Biobank after excluding (<18.5 kg/m^2^) participants at baseline, Table S9: Longitudinal associations between type-specific alcohol consumption and general adiposity at follow-up in the UK Biobank adjusting with daily energy intake as a dietary proxy, Table S10: Longitudinal associations between type-specific alcohol consumption and central adiposity at follow-up in the UK Biobank after excluding underweight (<18.5 kg/m^2^) participants at baseline, Table S11: The associations of total alcohol consumption and individual type of alcoholic drink with incident overweight (BMI ≥ 25 kg/m^2^) and obesity (≥30 kg/m^2^) by adjusting with daily energy intake as a dietary proxy, Table S12: The associations of total alcohol consumption with incident overweight (BMI ≥ 25 kg/m^2^) and obesity (≥30 kg/m^2^) after excluding non-current drinkers.
