# Supplementary material for: Alcohol Consumption and Adiposity: A Longitudinal Analysis of 45,399 UK Biobank Participants
Source: Int J Environ Res Public Health. 2022 Sep 21;19(19):11945. doi: 10.3390/ijerph191911945 (PMC9565835; doi:10.3390/ijerph191911945)
Supplement: Supplementary file 1 [file ijerph-19-11945-s001.zip › ijerph-1903195-supplementary.pdf]

Table S1. Alcohol consumption status change of the UK Biobank participants

UK Guidelines based alcohol consumption status change	Frequency (n)	Percent (%)	Cumulative Frequency (n)	Cumulative Percent (%)
Stable never drinkers	931	2.27	20652	2.3
Stable previous drinkers	636	1.55	30839	3.8
Stable occasional drinkers	5706	13.89	26358	17.7
Stable within guideline drinkers	10227	24.9	41066	42.6
Stable double the guidelines drinkers	5355	13.04	19721	55.7
Stable over double the guideline drinkers	3845	9.36	30203	**65.0**
Become previous drinkers	647	1.58	647	66.6
Decrease in alcohol consumption status	8714	21.22	9361	87.8
Increase in alcohol consumption status	5005	12.19	14366	100

Table S2. Longitudinal associations between baseline alcohol consumption and general adiposity at follow up in the UK Biobank participants without missing adiposity outcomes (n = 17,979)

	BMI				BF%			
	Model 1		Model 2		Model 1		Model 2	
	Coefficient	95% CI	Coefficient	95% CI	Coefficient	95% CI	Coefficient	95% CI
**Women**								
**Quartile 1**	0.17	-0.07, 0.4	0.17	-0.07, 0.4	**0.63**	**0.25, 1.02**	**0.61**	**0.22, 0.99**
**Quartile 2**	0.03	-0.21, 0.27	0.04	-0.19, 0.28	0.39	0, 0.78	0.37	-0.02, 0.76
**Quartile 3**	-0.04	-0.27, 0.19	-0.03	-0.26, 0.2	0.34	-0.04, 0.72	0.31	-0.07, 0.7
**Quartile 4**	0.04	-0.19, 0.27	0.04	-0.2, 0.27	**0.44**	**0.06, 0.82**	**0.39**	**0.01, 0.78**
**Men**								
**Quartile 1**	-0.18	-0.41, 0.04	-0.17	-0.4, 0.06	-0.24	-0.65, 0.17	-0.23	-0.64, 0.19
**Quartile 2**	-0.24	-0.47, -0.02	-0.22	-0.44, 0.01	-0.32	-0.73, 0.09	-0.29	-0.7, 0.12
**Quartile 3**	**-0.26**	**-0.48, -0.03**	**-0.23**	**-0.46, -0.01**	-0.38	-0.79, 0.03	-0.36	-0.77, 0.05
**Quartile 4**	-0.09	-0.32, 0.13	-0.08	-0.31, 0.14	-0.02	-0.43, 0.39	-0.06	-0.48, 0.35

Generalised linear model coefficient; mean differences (in risk factor values) between the reference category (non-current drinker) and each of the other alcohol consumption categories.
Alcohol consumption units by quartile: for women, 1st quartile: ≤1.3 unit; 2nd quartile: <6.9 unit; 3rd quartile: <14.3 unit; 4th quartile: ≥14.3 unit; for men, 1st quartile: ≤6.5 unit; 2nd quartile: <15.9 unit; 3rd quartile: <29.2 unit; 4th quartile: ≥29.2 unit.
Model 1 is adjusted for baseline age, baseline specific adiposity marker, and follow up time (yrs). Model 2 is additionally adjusted for Townsend Deprivation Index, smoking status, CVD/cancer history, sleep duration.
Body mass index (BMI) = Weight (kg)/height (m^2). BF% was measured by bioimpedance using the Tanita BC-418MA device (Tanita, Tokyo, Japan).
Bold font indicates statistical significance ($p<0.05$).

Table S3. Longitudinal associations between alcohol consumption and general adiposity at follow-up in the UK Biobank after excluding underweight (<18.5 kg/m²) participants at baseline

	BMI (n = 40,517)						BF% (n = 18,397)					
	**Model 1**		**Model 2**		**Model 3**		**Model 1**		**Model 2**		**Model 3**	
	**Coefficient**	**95% CI**	**Coefficient**	**95% CI**	**Coefficient**	**95% CI**	**Coefficient**	**95% CI**	**Coefficient**	**95% CI**	**Coefficient**	**95% CI**
**Women**												
**Quartile 1**	0.05	-0.12, 0.22	0.05	-0.11, 0.22	0.05	-0.11, 0.22	**0.59**	**0.21, 0.97**	**0.57**	**0.19, 0.95**	**0.57**	**0.19, 0.95**
**Quartile 2**	-0.09	-0.25, 0.08	-0.07	-0.24, 0.1	-0.07	-0.24, 0.1	**0.41**	**0.03, 0.8**	**0.4**	**0.01, 0.78**	**0.4**	**0.01, 0.79**
**Quartile 3**	-0.14	-0.3, 0.03	-0.12	-0.29, 0.04	-0.12	-0.29, 0.04	0.35	-0.03, 0.72	0.32	-0.05, 0.7	0.32	-0.05, 0.7
**Quartile 4**	-0.1	-0.27, 0.06	-0.11	-0.27, 0.06	-0.11	-0.27, 0.06	**0.44**	**0.06, 0.81**	**0.4**	**0.02, 0.78**	**0.4**	**0.02, 0.78**
**Men**												
**Quartile 1**	-0.09	-0.25, 0.07	-0.07	-0.23, 0.09	-0.07	-0.23, 0.09	-0.25	-0.66, 0.15	-0.25	-0.66, 0.16	-0.26	-0.66, 0.15
**Quartile 2**	-0.15	-0.3, 0.01	-0.1	-0.26, 0.05	-0.11	-0.27, 0.05	-0.32	-0.72, 0.09	-0.31	-0.71, 0.1	-0.31	-0.71, 0.1
**Quartile 3**	-0.16	-0.31, 0	-0.12	-0.28, 0.04	-0.13	-0.29, 0.03	-0.38	-0.79, 0.02	-0.38	-0.79, 0.02	-0.39	-0.8, 0.01
**Quartile 4**	0.04	-0.12, 0.2	0.04	-0.12, 0.2	0.03	-0.13, 0.19	-0.08	-0.48, 0.32	-0.13	-0.54, 0.28	-0.14	-0.55, 0.27

Generalised linear model coefficient; mean differences (in risk factor values) between the reference category (current non- drinker) and each of the other alcohol consumption categories.
Alcohol consumption units by quartile: for women, 1st quartile: ≤1.3 unit; 2nd quartile: <6.9 unit; 3rd quartile: <14.3 unit; 4th quartile: ≥14.3 unit; for men, 1st quartile: ≤6.5 unit; 2nd quartile: <15.9 unit; 3rd quartile: <29.2 unit; 4th quartile: ≥29.2 unit.
Model 1 is adjusted for baseline age, specific adiposity marker, and follow up time. Model 2 is further adjusted for Townsend Deprivation Index, smoking status, major illness, sleep duration (hrs/night). Model 3 is further adjusted for physical activity, sitting time, daily vegetable and fruit consumption.
Body mass index (BMI) = Weight (kg)/height (m2). BF% was measured by bioimpedance using the Tanita BC-418MA device (Tanita, Tokyo, Japan).
Bold font indicates statistical significance ($p<0.05$).

Table S4. Longitudinal associations between alcohol consumption and general adiposity at follow-up in the UK Biobank adjusting with daily energy intake as a dietary proxy

	**General Adiposity**				**Central Adiposity**			
	**BMI (n = 17,825)**		**BF% (n = 8,208)**		**WC (n = 17,860)**		**WHR (n = 8,188)**	
	**Coefficient**	**95% CI**	**Coefficient**	**95% CI**	**Coefficient**	**95% CI**	**Coefficient**	**95% CI**
**Women**								
Quartile 1	0.01	-0.24, 0.26	0.76	0.18, 1.34	0.38	-0.51, 1.26	0.007	-0.003, 0.017
Quartile 2	-0.12	-0.37, 0.12	0.5	-0.07, 1.07	-0.7	-1.57, 0.17	0.001	-0.009, 0.01
Quartile 3	-0.16	-0.41, 0.08	0.4	-0.16, 0.97	-0.72	-1.58, 0.14	0.002	-0.008, 0.012
Quartile 4	-0.13	-0.37, 0.12	0.46	-0.11, 1.03	-0.37	-1.24, 0.49	0.003	-0.007, 0.012
**Men**								
Quartile 1	-0.08	-0.32, 0.16	-0.24	-0.85, 0.38	-0.18	-1.17, 0.8	0.002	-0.01, 0.013
Quartile 2	-0.13	-0.37, 0.11	-0.35	-0.96, 0.26	-0.42	-1.39, 0.55	0	-0.011, 0.011
Quartile 3	-0.14	-0.38, 0.09	-0.34	-0.95, 0.27	-0.34	-1.31, 0.63	0.001	-0.01, 0.012
Quartile 4	0.04	-0.2, 0.28	-0.14	-0.76, 0.48	0.16	-0.83, 1.15	0.005	-0.007, 0.016

eneralised linear model coefficient; mean differences (in risk factor values) between the reference category (current non-drinker) and each of the other alcohol consumption categories.
Alcohol consumption units by quartile: for women, 1st quartile: ≤1.3 unit; 2nd quartile: <6.9 unit; 3rd quartile: <14.3 unit; 4th quartile: ≥14.3 unit; for men, 1st quartile: ≤6.5 unit; 2nd quartile: <15.9 unit; 3rd quartile: <29.2 unit; 4th quartile: ≥29.2 unit.
Model is adjusted for baseline age, specific adiposity marker, follow up time (yrs), Townsend Deprivation Index, smoking status, major illness, sleep duration, PA, sitting time, and daily energy intake.
Body mass index (BMI) = Weight (kg)/height (m2). BF% was measured by bioimpedance using the Tanita BC-418MA device (Tanita, Tokyo, Japan). WC and hip circumference were measured by using flexible plastic tape with the participant in the resting-standing position by a trained professional.

Table S5. Longitudinal associations between alcohol consumption at baseline and general adiposity at follow-up in the UK Biobank after excluding non-current drinkers

	BMI (n = 40,517)						BF% (n = 18,397)					
	Model 1		Model 2		Model 3		Model 1		Model 2		Model 3	
	Coefficient	95% CI	Coefficient	95% CI	Coefficient	95% CI	Coefficient	95% CI	Coefficient	95% CI	Coefficient	95% CI
**Women**												
**Quartile 2**	-0.05	-0.14, 0.03	-0.04	-0.12, 0.05	**-0.12**	**-0.23, -0.02**	-0.17	-0.42, 0.08	-0.18	-0.43, 0.07	-0.17	-0.42, 0.08
**Quartile 3**	-0.06	-0.15, 0.02	-0.06	-0.14, 0.03	**-0.17**	**-0.27, -0.07**	**-0.25**	**-0.48, -0.01**	**-0.26**	**-0.49, -0.02**	**-0.26**	**-0.49, -0.02**
**Quartile 4**	0.13	0.04, 0.21	0.1	0.02, 0.19	**-0.16**	**-0.26, -0.06**	-0.16	-0.4, 0.07	-0.19	-0.42, 0.05	-0.18	-0.42, 0.06
**Men**												
**Quartile 2**	**-0.12**	**-0.23, -0.02**	**-0.12**	**-0.23, -0.02**	-0.04	-0.12, 0.04	-0.07	-0.28, 0.15	-0.06	-0.27, 0.15	-0.06	-0.27, 0.16
**Quartile 3**	**-0.17**	**-0.27, -0.07**	**-0.17**	**-0.27, -0.07**	-0.06	-0.14, 0.02	-0.14	-0.35, 0.07	-0.15	-0.36, 0.06	-0.15	-0.36, 0.06
**Quartile 4**	**-0.16**	**-0.26, -0.06**	**-0.16**	**-0.26, -0.06**	**0.09**	**0.01, 0.18**	0.17	-0.05, 0.38	0.11	-0.1, 0.33	0.11	-0.11, 0.33
	WC (n = 40,571)						WHR (n = 18,383)					
	Model 1		Model 2		Model 3		Model 1		Model 2		Model 3	
	Coefficient	95% CI	Coefficient	95% CI	Coefficient	95% CI	Coefficient	95% CI	Coefficient	95% CI	Coefficient	95% CI
**Women**												
**Quartile 2**	**-0.91**	**-1.28, -0.55**	**-0.87**	**-1.24, -0.51**	**-0.86**	**-1.22, -0.5**	**-0.006**	**-0.01, -0.002**	**-0.006**	**-0.01, -0.001**	**-0.006**	**-0.01, -0.001**
**Quartile 3**	**-0.95**	**-1.3, -0.61**	**-0.91**	**-1.26, -0.57**	**-0.9**	**-1.25, -0.56**	-0.003	-0.007, 0.001	-0.003	-0.007, 0.001	-0.003	-0.007, 0.001
**Quartile 4**	**-0.76**	**-1.1, -0.41**	**-0.78**	**-1.13, -0.44**	**-0.77**	**-1.12, -0.42**	-0.003	-0.007, 0.001	-0.004	-0.008, 0	-0.004	-0.008, 0
**Men**												
**Quartile 2**	**-0.35**	**-0.68, -0.02**	-0.3	-0.63, 0.03	-0.28	-0.61, 0.04	-0.002	-0.006, 0.002	-0.002	-0.006, 0.002	-0.002	-0.005, 0.002
**Quartile 3**	-0.25	-0.58, 0.08	-0.24	-0.57, 0.09	-0.23	-0.56, 0.1	-0.002	-0.006, 0.001	-0.002	-0.006, 0.001	-0.002	-0.006, 0.002
**Quartile 4**	**0.5**	**0.17, 0.84**	**0.4**	**0.06, 0.74**	**0.41**	**0.07, 0.75**	0.003	-0.001, 0.006	0.002	-0.002, 0.006	0.002	-0.002, 0.006

Generalised linear model coefficient; mean differences (in risk factor values) between the reference category (current non- drinker) and each of the other alcohol consumption categories.
Alcohol consumption units by for women, 1st quartile: ≤1.3 unit; 2nd quartile: <6.9 unit; 3rd quartile: <14.3 unit; 4th quartile: ≥14.3 unit; for men, 1st quartile: ≤6.5 unit; 2nd quartile: <15.9 unit; 3rd quartile: <29.2 unit; 4th quartile: ≥29.2 unit.
Model 1 is adjusted for baseline age, specific adiposity marker, and follow up time. Model 2 is further adjusted for Townsend Deprivation Index, smoking status, major illness, sleep duration (hrs/night). Model 3 is further adjusted for physical activity, sitting time, daily vegetable and fruit consumption.

Body mass index (BMI) = Weight (kg)/height (m2). BF% was measured by bioimpedance using the Tanita BC-418MA device (Tanita, Tokyo, Japan). WC and hip circumference were measured by using flexible plastic tape with the participant in the resting-standing position by a trained professional.
Bold font indicates statistical significance (p<0.05).

Table S6. Longitudinal associations between baseline alcohol consumption and central adiposity at follow-up in the UK Biobank participants without missing adiposity outcomes (n = 17,979)

	WC				WHR			
	Model 1		Model 2		Model 1		Model 2	
	Coefficient	95% CI	Coefficient	95% CI	Coefficient	95% CI	Coefficient	95% CI
**Women**								
**Quartile 1**	0.73	-0.08, 1.54	0.75	-0.06, 1.56	0.004	-0.002, 0.011	0.004	-0.002, 0.011
**Quartile 2**	-0.25	-1.06, 0.57	-0.19	-1.01, 0.64	-0.002	-0.008, 0.005	-0.002	-0.008, 0.005
**Quartile 3**	-0.38	-1.18, 0.42	-0.33	-1.13, 0.47	0	-0.006, 0.007	0	-0.006, 0.006
**Quartile 4**	0.08	-0.72, 0.88	0.08	-0.73, 0.88	0.001	-0.005, 0.008	0	-0.006, 0.006
**Men**								
**Quartile 1**	-0.52	-1.43, 0.39	-0.46	-1.37, 0.45	0.003	-0.004, 0.01	0.004	-0.004, 0.011
**Quartile 2**	-0.89	-1.8, 0.01	-0.79	-1.69, 0.12	0.001	-0.006, 0.008	0.002	-0.005, 0.009
**Quartile 3**	-0.84	-1.74, 0.06	-0.73	-1.64, 0.17	0	-0.007, 0.008	0.001	-0.006, 0.008
**Quartile 4**	-0.14	-1.04, 0.77	-0.11	-1.02, 0.8	0.005	-0.002, 0.013	0.005	-0.002, 0.012

Generalised linear model coefficient; mean differences (in risk factor values) between the reference category (non-current drinker) and each of the other alcohol consumption categories.
Alcohol consumption units by quartile: for women, 1st quartile: ≤1.3 unit; 2nd quartile: <6.9 unit; 3rd quartile: <14.3 unit; 4th quartile: ≥14.3 unit; for men, 1st quartile: ≤6.5 unit; 2nd quartile: <15.9 unit; 3rd quartile: <29.2 unit; 4th quartile: ≥29.2 unit.
Model 1 is adjusted for baseline age, baseline specific adiposity marker, and follow up time (yrs). Model 2 is additionally adjusted for Townsend Deprivation Index, smoking status, CVD/cancer history, sleep duration.
WC and hip circumference were measured by using flexible plastic tape with the participant in the resting-standing position by a trained professional.

Table S7. Longitudinal associations between alcohol consumption and central adiposity at follow-up in the UK Biobank after excluding underweight (<18.5 kg/m^2) participants at baseline

	WC (n = 40,571)						WHR (n = 18,383)					
	Model 1		Model 2		Model 3		Model 1		Model 2		Model 3	
	Coefficient	95% CI	Coefficient	95% CI	Coefficient	95% CI	Coefficient	95% CI	Coefficient	95% CI	Coefficient	95% CI
**Women**												
**Quartile 1**	0.47	-0.11, 1.05	0.49	-0.09, 1.07	0.48	-0.09, 1.06	0.004	-0.002, 0.011	0.004	-0.002, 0.011	0.004	-0.002, 0.011
**Quartile 2**	-0.47	-1.05, 0.11	-0.4	-0.98, 0.18	-0.4	-0.97, 0.18	-0.001	-0.008, 0.005	-0.001	-0.008, 0.005	-0.001	-0.008, 0.005
**Quartile 3**	-0.51	-1.08, 0.05	-0.45	-1.02, 0.12	-0.45	-1.01, 0.12	0.001	-0.005, 0.007	0.001	-0.005, 0.007	0.001	-0.005, 0.007
**Quartile 4**	-0.33	-0.9, 0.24	-0.33	-0.9, 0.24	-0.32	-0.89, 0.25	0.002	-0.005, 0.008	0.001	-0.006, 0.007	0.001	-0.006, 0.007
**Men**												
**Quartile 1**	-0.12	-0.76, 0.51	-0.04	-0.67, 0.6	-0.06	-0.69, 0.58	0.003	-0.004, 0.011	0.004	-0.003, 0.011	0.004	-0.003, 0.011
**Quartile 2**	-0.48	-1.1, 0.15	-0.34	-0.97, 0.29	-0.35	-0.97, 0.28	0.001	-0.006, 0.008	0.002	-0.005, 0.009	0.003	-0.005, 0.01
**Quartile 3**	-0.38	-1, 0.25	-0.28	-0.91, 0.35	-0.29	-0.91, 0.34	0.001	-0.006, 0.008	0.002	-0.006, 0.009	0.002	-0.005, 0.009
**Quartile 4**	0.38	-0.25, 1.01	0.36	-0.27, 0.99	0.35	-0.28, 0.98	0.006	-0.001, 0.013	0.005	-0.002, 0.013	0.006	-0.001, 0.013

Generalised linear model coefficient; mean differences (in risk factor values) between the reference category (current non- drinker) and each of the other alcohol consumption categories.
Alcohol consumption units by for women, 1st quartile: ≤1.3 unit; 2nd quartile: <6.9 unit; 3rd quartile: <14.3 unit; 4th quartile: ≥14.3 unit; for men, 1st quartile: ≤6.5 unit; 2nd quartile: <15.9 unit; 3rd quartile: <29.2 unit; 4th quartile: ≥29.2 unit.
Model 1 is adjusted for baseline age, specific adiposity marker, and follow up time. Model 2 is further adjusted for Townsend Deprivation Index, smoking status, major illness, sleep duration (hrs/night). Model 3 is further adjusted for physical activity, sitting time, daily vegetable and fruit consumption.
WC and hip circumference were measured by using flexible plastic tape with the participant in the resting-standing position by a trained professional.

Table S8. Longitudinal associations between type specific alcohol consumption and general adiposity at follow-up in the UK Biobank after excluding (<18.5 kg/m^2) participants at baseline

	Model 1		Model 2		Model 3	
	**Coefficient**	**95% CI**	**Coefficient**	**95% CI**	**Coefficient**	**95% CI**
BMI (n = 40,517)						
**Women**						
Red wine	**-0.11**	**-0.17, -0.06**	**-0.1**	**-0.15, -0.04**	**-0.1**	**-0.16, -0.04**
Champagne	**-0.1**	**-0.16, -0.04**	**-0.08**	**-0.14, -0.03**	**-0.08**	**-0.14, -0.03**
Beer	-0.04	-0.11, 0.03	-0.04	-0.12, 0.03	-0.04	-0.11, 0.03
Spirits	0.06	0, 0.13	0.05	-0.01, 0.12	0.05	-0.01, 0.12
Fortified wine	0	-0.09, 0.1	0.02	-0.08, 0.12	0.02	-0.08, 0.12
**Men**						
Red wine	**-0.09**	**-0.14, -0.04**	**-0.06**	**-0.11, -0.01**	**-0.06**	**-0.11, -0.01**
Champagne	-0.07	-0.12, -0.02	-0.05	-0.09, 0	-0.05	-0.09, 0
Beer	-0.02	-0.07, 0.03	-0.02	-0.07, 0.03	-0.03	-0.08, 0.02
Spirits	0.04	-0.01, 0.09	0.03	-0.02, 0.08	0.03	-0.02, 0.08
Fortified wine	-0.02	-0.11, 0.06	-0.01	-0.09, 0.08	-0.01	-0.09, 0.08
BF% (n = 18,397)						
**Women**						
Red wine	-0.07	-0.2, 0.07	-0.07	-0.2, 0.07	-0.06	-0.2, 0.07
Champagne	-0.04	-0.18, 0.09	-0.04	-0.18, 0.09	-0.04	-0.17, 0.1
Beer	-0.1	-0.27, 0.08	-0.1	-0.28, 0.07	-0.09	-0.27, 0.08
Spirits	0.15	0, 0.31	0.14	-0.02, 0.29	0.13	-0.02, 0.29
Fortified wine	-0.02	-0.24, 0.2	-0.02	-0.24, 0.2	-0.02	-0.24, 0.2
**Men**						
Red wine	**-0.18**	**-0.3, -0.05**	**-0.17**	**-0.29, -0.04**	**-0.15**	**-0.28, -0.03**
Champagne	**-0.13**	**-0.25, -0.01**	-0.11	-0.23, 0.01	-0.1	-0.22, 0.02
Beer	0.03	-0.1, 0.15	0.02	-0.11, 0.14	0.02	-0.11, 0.14
Spirits	-0.03	-0.16, 0.09	-0.05	-0.18, 0.08	-0.05	-0.17, 0.08
Fortified wine	-0.05	-0.27, 0.16	-0.03	-0.25, 0.18	-0.02	-0.24, 0.19

Generalised linear model coefficient; mean differences (in risk factor values) between participants who did not consume the relevant alcohol type (the referent) and participants who reported consuming the relevant alcohol type.
Model 1 is adjusted for baseline age, specific adiposity marker, and follow up time. Model 2 is further adjusted for Townsend Deprivation Index, smoking status, major illness, sleep duration (hrs/night). Model 3 is further adjusted for physical activity, sitting time, daily vegetable and fruit consumption.
Body mass index (BMI) = Weight (kg)/height (m2). BF% was measured by bioimpedance using the Tanita BC-418MA device (Tanita, Tokyo, Japan).
Bold font indicates statistical significance ($p<0.05$).

Table S9. Longitudinal associations between type specific alcohol consumption and general adiposity at follow-up in the UK Biobank adjusting with daily energy intake as a dietary proxy

	Coefficient	95% CI		Coefficient	95% CI
**General Adiposity**					
BMI (n = 17,825)			*BF% (n = 8,208)*		
**Women**			**Women**		
Red wine	**-0.11**	**-0.2, -0.03**	Red wine	-0.11	-0.32, 0.09
Champagne	-0.05	-0.13, 0.04	Champagne	-0.02	-0.22, 0.18
Beer	-0.03	-0.13, 0.07	Beer	-0.2	-0.44, 0.05
Spirits	0.07	-0.02, 0.17	Spirits	0.04	-0.19, 0.27
Fortified wine	0.05	-0.08, 0.19	Fortified wine	0	-0.32, 0.32
**Men**			**Men**		
Red wine	-0.06	-0.14, 0.01	Red wine	**-0.22**	**-0.41, -0.03**
Champagne	0	-0.07, 0.07	Champagne	-0.09	-0.27, 0.09
Beer	-0.04	-0.12, 0.03	Beer	-0.03	-0.22, 0.16
Spirits	0.04	-0.03, 0.11	Spirits	-0.04	-0.23, 0.14
Fortified wine	0.05	-0.07, 0.18	Fortified wine	0.03	-0.28, 0.34
**Central Adiposity**					
WC (n = 17,860)			*WHR (n = 8,188)*		
**Women**			**Women**		
Red wine	**-0.71**	**-1.01, -0.41**	Red wine	-0.003	-0.007, 0
Champagne	**-0.44**	**-0.73, -0.14**	Champagne	-0.003	-0.007, 0
Beer	-0.19	-0.55, 0.17	Beer	0.001	-0.003, 0.005
Spirits	0.23	-0.11, 0.57	Spirits	-0.002	-0.006, 0.002
Fortified wine	-0.12	-0.62, 0.37	Fortified wine	-0.001	-0.007, 0.004
**Men**			**Men**		
Red wine	**-0.33**	**-0.64, -0.03**	Red wine	-0.002	-0.006, 0.001
Champagne	-0.1	-0.38, 0.18	Champagne	-0.001	-0.005, 0.002
Beer	**-0.44**	**-0.74, -0.13**	Beer	0.001	-0.003, 0.004
Spirits	0.22	-0.08, 0.51	Spirits	0.004	0, 0.007
Fortified wine	0.36	-0.13, 0.86	Fortified wine	0.001	-0.004, 0.007

Generalised linear model coefficient; mean differences (in risk factor values) between participants who did not consume the relevant alcohol type (the referent) and participants who reported consuming the relevant alcohol type.
Model 1 is adjusted for baseline age, specific adiposity marker, and follow up time (yrs). Model is adjusted for baseline age, specific adiposity marker, follow up time (yrs), Townsend Deprivation Index, smoking status, major illness, sleep duration, PA, sitting time, and daily energy intake.
Body mass index (BMI) = Weight (kg)/height (m2). BF% was measured by bioimpedance using the Tanita BC-418MA device (Tanita, Tokyo, Japan). WC and hip circumference were measured by using flexible plastic tape with the participant in the resting-standing position by a trained professional.
Bold font indicates statistical significance ($p<0.05$).

Table S10. Longitudinal associations between type specific alcohol consumption and central adiposity at follow-up in the UK Biobank after excluding underweight (<18.5 kg/m^2) participants at baseline

	**Model 1**		**Model 2**		**Model 3**	
	**Coefficient**	**95% CI**	**Coefficient**	**95% CI**	**Coefficient**	**95% CI**
WC (n = 40,571)						
**Women**						
Red wine	**-0.67**	**-0.86, -0.47**	**-0.62**	**-0.82, -0.43**	**-0.6**	**-0.79, -0.4**
Champagne	**-0.56**	**-0.76, -0.37**	**-0.51**	**-0.7, -0.31**	**-0.5**	**-0.69, -0.3**
Beer	**-0.37**	**-0.62, -0.13**	**-0.39**	**-0.63, -0.14**	**-0.37**	**-0.61, -0.12**
Spirits	0.29	0.07, 0.51	0.27	0.05, 0.5	0.26	0.04, 0.48
Fortified wine	-0.26	-0.6, 0.08	-0.21	-0.55, 0.13	-0.19	-0.53, 0.15
**Men**						
Red wine	**-0.4**	**-0.6, -0.21**	**-0.32**	**-0.51, -0.12**	**-0.28**	**-0.47, -0.08**
Champagne	**-0.34**	**-0.53, -0.15**	**-0.25**	**-0.44, -0.06**	**-0.23**	**-0.42, -0.04**
Beer	**-0.29**	**-0.49, -0.09**	**-0.29**	**-0.49, -0.09**	**-0.29**	**-0.49, -0.09**
Spirits	**0.24**	**0.05, 0.44**	**0.22**	**0.02, 0.42**	**0.22**	**0.02, 0.41**
Fortified wine	0.01	-0.33, 0.35	0.08	-0.26, 0.42	0.1	-0.24, 0.43
WHR (n = 18,383)						
**Women**						
Red wine	-0.003	-0.005, 0	-0.003	-0.005, 0	-0.002	-0.005, 0
Champagne	**-0.003**	**-0.005, -0.001**	-0.003	-0.005, 0	-0.003	-0.005, 0
Beer	0	-0.003, 0.003	-0.001	-0.004, 0.002	0	-0.003, 0.003
Spirits	0.002	-0.001, 0.004	0.001	-0.001, 0.004	0.001	-0.001, 0.004
Fortified wine	-0.001	-0.005, 0.003	0	-0.004, 0.003	0	-0.004, 0.003
**Men**						
Red wine	-0.002	-0.004, 0.001	-0.001	-0.003, 0.001	-0.001	-0.003, 0.001
Champagne	-0.002	-0.004, 0	-0.001	-0.003, 0.001	-0.001	-0.003, 0.001
Beer	-0.001	-0.003, 0.002	-0.001	-0.003, 0.001	0	-0.003, 0.002
Spirits	0.003	0, 0.005	0.003	0, 0.005	0.003	0, 0.005
Fortified wine	0	-0.003, 0.004	0.001	-0.003, 0.005	0.001	-0.003, 0.005

Generalised linear model coefficient; mean differences (in risk factor values) between participants who did not consume the relevant alcohol type (the referent) and participants who reported consuming the relevant alcohol type.
Model 1 is adjusted for baseline age, specific adiposity marker, and follow up time. Model 2 is further adjusted for Townsend Deprivation Index, smoking status, major illness, sleep duration (hrs/night). Model 3 is further adjusted for physical activity, sitting time, daily vegetable and fruit consumption.
WC and hip circumference were measured by using flexible plastic tape with the participant in the resting-standing position by a trained professional.
Bold font indicates statistical significance ($p<0.05$).

Table S11. The associations of total alcohol consumption and individual type of alcoholic drink with incident overweight (BMI ≥25 kg/m[2]) and obesity (≥ 30 kg/m[2]) by adjusting with daily energy intake as a dietary proxy

	**Incident overweight (BMI ≥25 kg/m[2])**				**Incident obesity (≥ 30 kg/m[2])**			
	**Women (case/total=574/4,399)**		**Men (431/2,680)**		**Women (397/7,598)**		**Men (340/6,964)**	
	**Odds Ratio**	**95% CI**	**Odds Ratio**	**95% CI**	**Odds Ratio**	**95% CI**	**Odds Ratio**	**95% CI**
Total alcohol consumption[a]								
Quartile 1	1.33	0.86, 2.04	0.67	0.4, 1.11	0.83	0.54, 1.27	0.81	0.47, 1.41
Quartile 2	1.04	0.68, 1.59	0.78	0.48, 1.29	**0.63**	**0.41, 0.97**	0.63	0.36, 1.1
Quartile 3	0.82	0.53, 1.25	0.88	0.53, 1.46	**0.57**	**0.37, 0.87**	0.66	0.38, 1.15
Quartile 4	0.98	0.64, 1.51	0.78	0.46, 1.32	**0.62**	**0.41, 0.95**	1.02	0.59, 1.75
Alcohol type b								
Red wine	0.83	0.68, 1.01	1	0.78, 1.28	**0.76**	**0.61, 0.96**	**0.67**	**0.52, 0.85**
Champagne	**0.81**	**0.67, 0.98**	0.95	0.76, 1.19	0.99	0.79, 1.24	0.88	0.69, 1.1
Beer	1.06	0.85, 1.31	0.94	0.74, 1.2	0.88	0.68, 1.14	0.81	0.63, 1.05
Spirits	1.22	0.99, 1.5	1.14	0.91, 1.44	**1.35**	**1.07, 1.69**	1.12	0.88, 1.41
Fortified wine	0.88	0.64, 1.22	0.87	0.59, 1.27	1	0.69, 1.46	1.07	0.72, 1.6

Multiple logistic regression model.
Alcohol consumption units by quartile: for women, 1st quartile: ≤1.3 unit; 2nd quartile: <6.9 unit; 3rd quartile: <14.3 unit; 4th quartile: ≥14.3 unit; for men, 1st quartile: ≤6.5 unit; 2nd quartile: <15.9 unit; 3rd quartile: <29.2 unit; 4th quartile: ≥29.2 unit. Model is adjusted for baseline age, Townsend Deprivation Index, smoking status, major illness, sleep duration, PA, sitting time, follow-up time and daily energy intake In type-specific analyses, models were further adjusted for total alcohol consumption .
[a] Non-current drinkers is the referent group.
[b] Participants who did not consume the relevant alcohol type is the referent group.
Bold font indicates statistical significance ($p<0.05$).

Table S12. The associations of total alcohol consumption with incident overweight (BMI ≥25 kg/m²) and obesity (≥ 30 kg/m²) after excluding non-current drinkers

	**Incident overweight (BMI ≥25 kg/m²)**				**Incident obesity (≥ 30 kg/m²)**			
	**Women (case/total=1,310/9,198)**		**Men (890/5,509)**		**Women (830/16,119)**		**Men (825/15,149)**	
	**Odds Ratio**	**95% CI**	**Odds Ratio**	**95% CI**	**Odds Ratio**	**95% CI**	**Odds Ratio**	**95% CI**
***Total alcohol consumption*[a]**								
Quartile 2	**0.73**	**0.61, 0.87**	1.03	0.84, 1.26	**0.65**	**0.53, 0.79**	0.86	0.7, 1.06
Quartile 3	**0.69**	**0.58, 0.81**	0.97	0.79, 1.2	**0.57**	**0.47, 0.69**	0.89	0.72, 1.09
Quartile 4	**0.78**	**0.66, 0.92**	1.24	0.99, 1.54	**0.65**	**0.54, 0.79**	1.18	0.96, 1.44

Multiple logistic regression model.
Alcohol consumption units by quartile: for women, 1st quartile: ≤1.3 unit; 2nd quartile: <6.9 unit; 3rd quartile: <14.3 unit; 4th quartile: ≥14.3 unit; for men, 1st quartile: ≤6.5 unit; 2nd quartile: <15.9 unit; 3rd quartile: <29.2 unit; 4th quartile: ≥29.2 unit. Model is adjusted for baseline age, Townsend Deprivation Index, smoking status, major illness, sleep duration, PA, sitting time, daily vegetable, follow-up time and fruit consumption In type-specific analyses, models were further adjusted for total alcohol consumption .
[a] The 1st quartile served as the referent group.
Bold font indicates statistical significance ($p<0.05$).

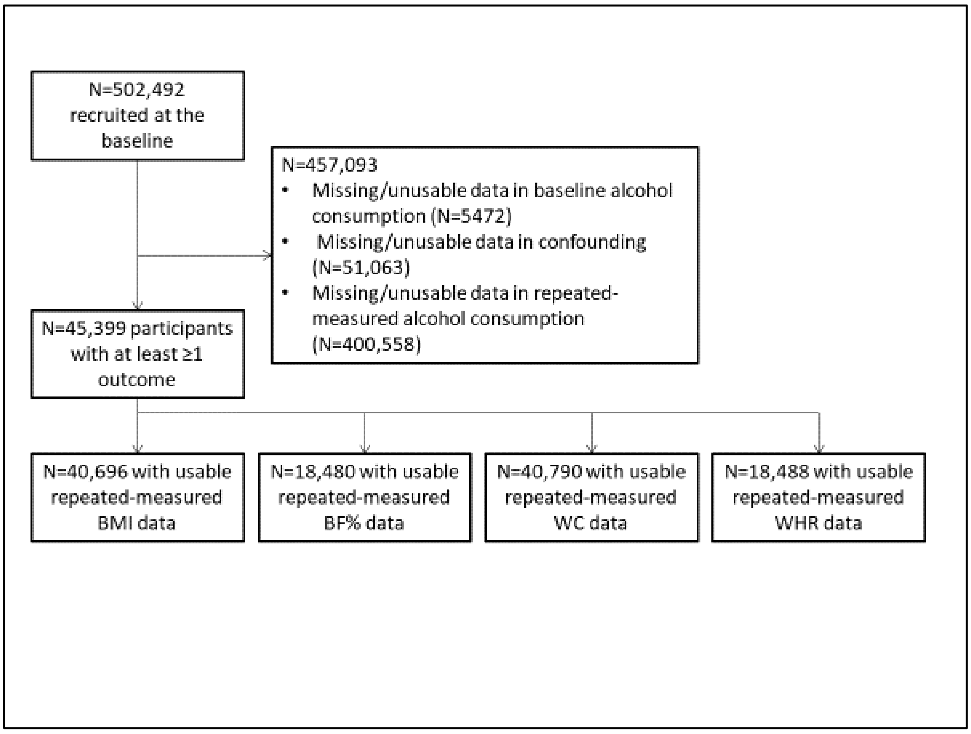

Figure S1. The flow chart of participants

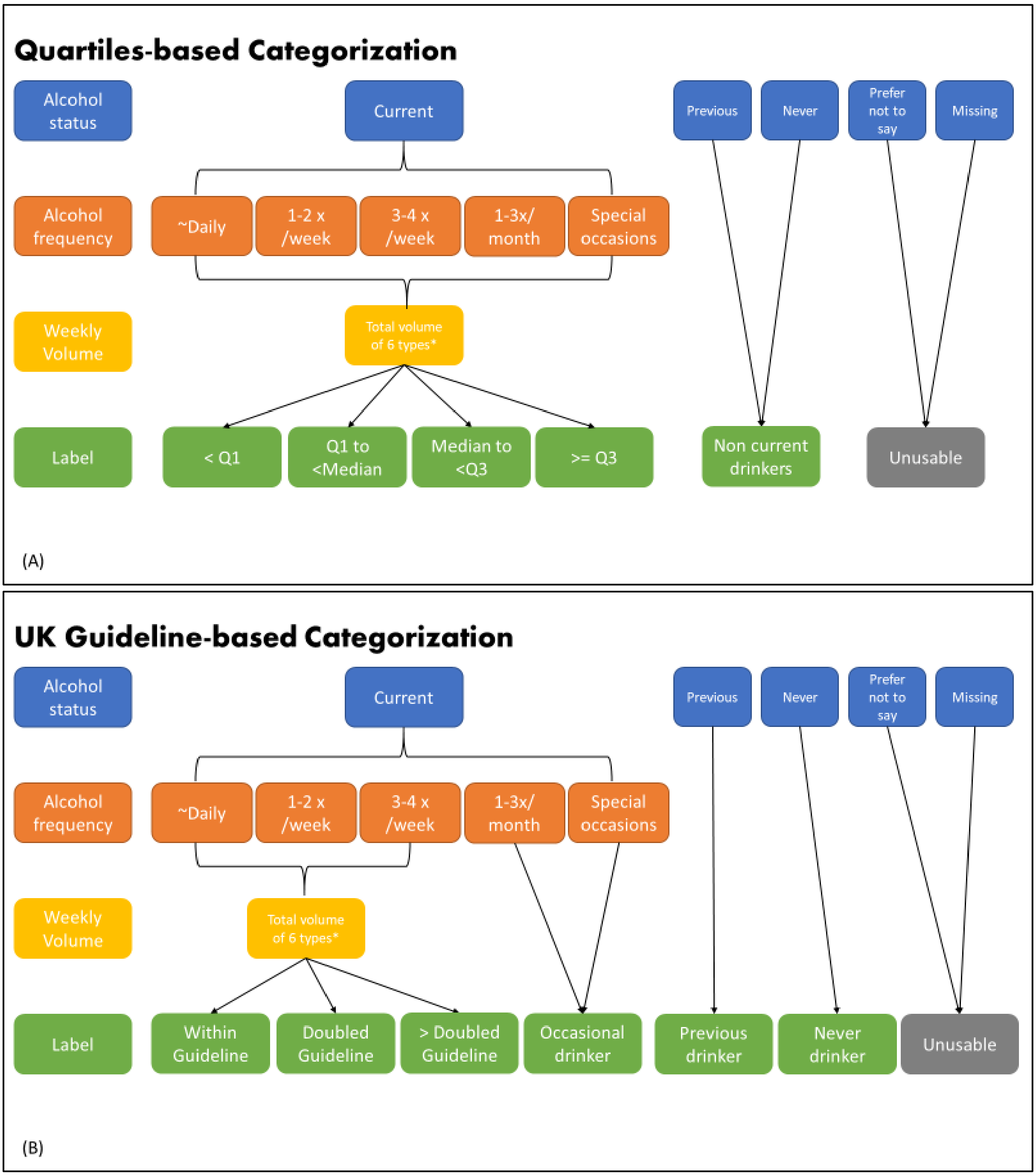

Figure S2. (A-B). The categorization of alcohol consumption

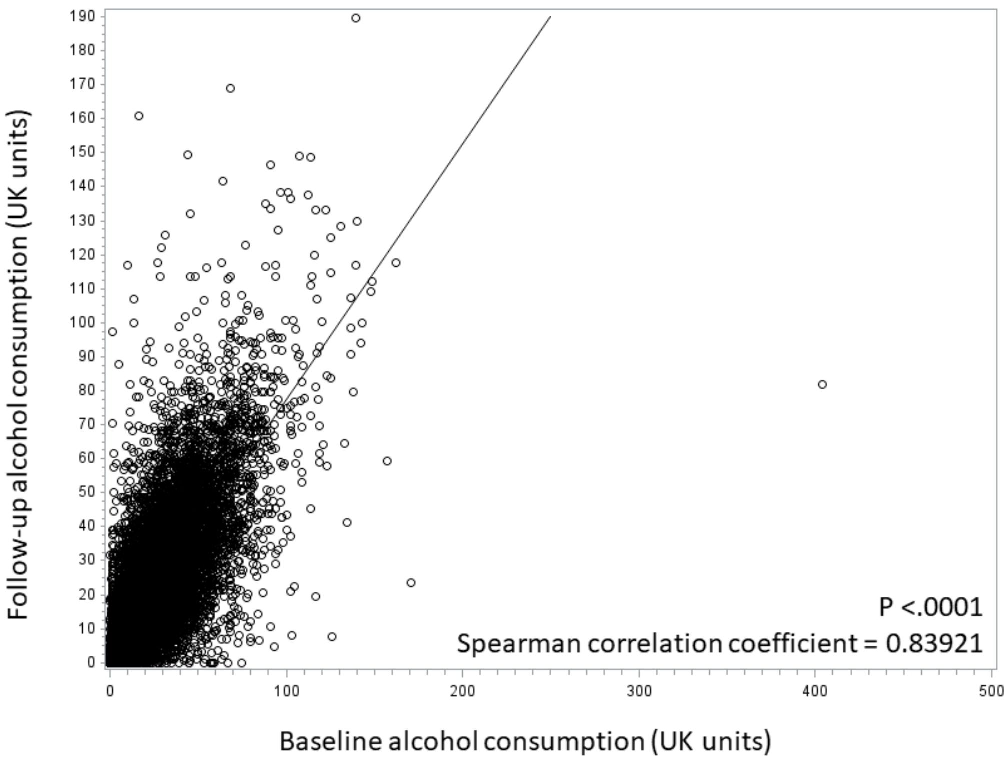

Figure S3. Correlation between baseline and follow up alcohol consumption

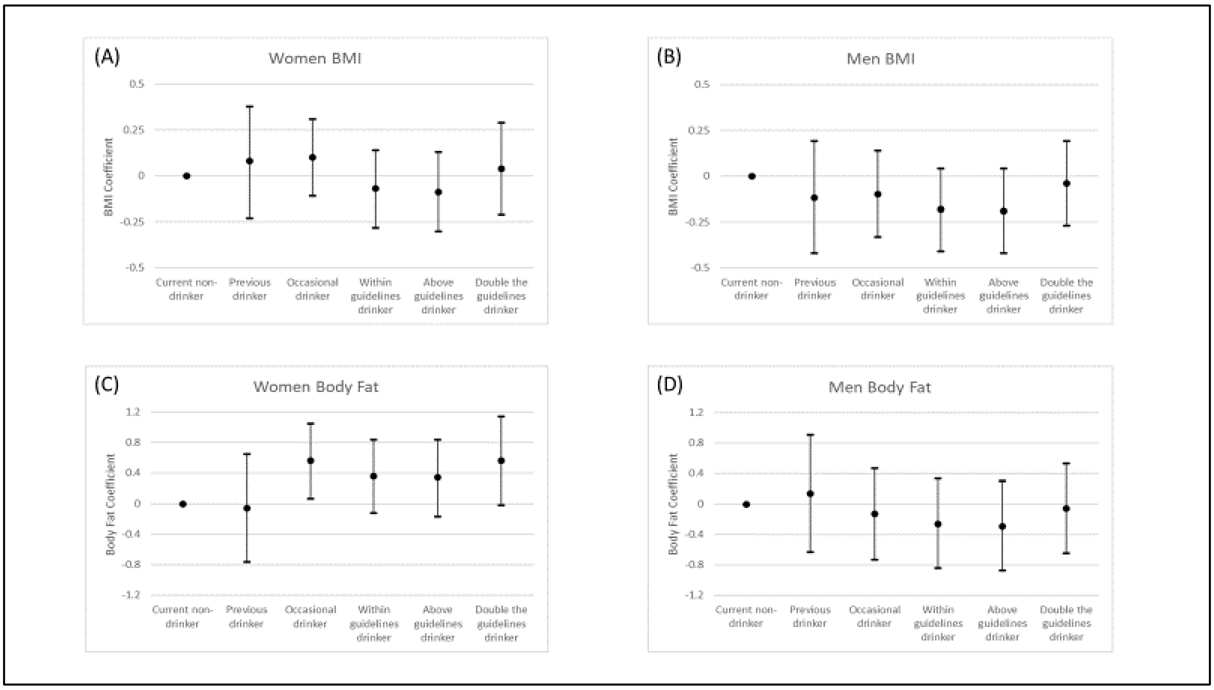

Figure S4. (A-D). Prospective associations between alternative alcohol consumption categorization and general adiposity by sex in the UK Biobank (Model 3). The coefficients were given in the y-axis for each adiposity marker and display the mean difference between the reference category (current non-drinkers) and the other consumption categories.

Model 3 is adjusted for baseline age, specific adiposity marker, follow up time (yrs), Townsend Deprivation Index, smoking status, major illness, sleep duration, PA, sitting time, daily vegetable and fruit consumption. Body mass index (BMI) = Weight (kg)/height (m2). BF% was measured by bioimpedance using the Tanita BC-418MA device (Tanita, Tokyo, Japan). Alcohol consumption units by the UK alcohol consumption guideline.

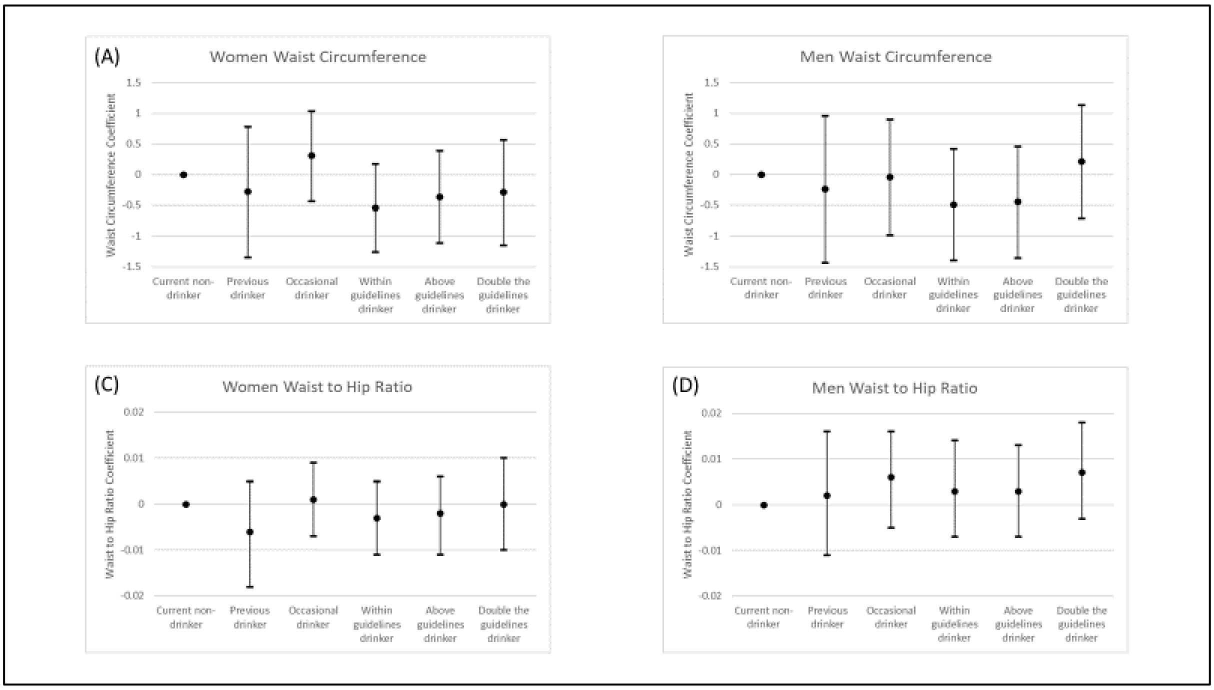

Figure S5 (A-D). Prospective associations between alternative alcohol consumption categorization and central adiposity by sex in the UK Biobank (Model 3). The coefficients were given in the y-axis for each adiposity marker and display the mean difference between the reference category (current non-drinkers) and the other consumption categories.

Model 3 is adjusted for baseline age, specific adiposity marker, follow up time (yrs), Townsend Deprivation Index, smoking status, major illness, sleep duration, PA, sitting time, daily vegetable and fruit consumption. WC and hip circumference were measured by using flexible plastic tape with the participant in the resting-standing position by a trained professional. Alcohol consumption units by the UK alcohol consumption guideline.
